# Evaluation of Two Fecal Occult Blood Tests for Detecting Non-Perforating Abomasal Lesions in Cattle

**DOI:** 10.3390/ani10122356

**Published:** 2020-12-09

**Authors:** Sara Lee Munch, Søren Saxmose Nielsen, Mogens Agerbo Krogh, Nynne Capion

**Affiliations:** 1 Department of Veterinary Clinical Sciences, University of Copenhagen, Højbakkegård Alle 5A, DK-2630 Taastrup, Denmark; slm@sund.ku.dk; 2 Department of Veterinary and Animal Sciences, University of Copenhagen, Grønnegårdsvej 8, DK-1870 Frederiksberg, Denmark; saxmose@sund.ku.dk; 3 Department of Animal Science, Aarhus University, Blichers Allé 20, DK-8830 Tjele, Denmark; mogenskrogh@anis.au.dk

**Keywords:** abomasal lesion, abomasal ulcer, bovine, dairy, diagnostic test, fecal occult blood

## Abstract

**Simple Summary:**

Abomasal lesions in dairy cattle are highly prevalent, but diagnosis of the non-penetrating lesions is a challenge. We performed one experiment to estimate the amount of blood detectable in feces using two different tests, one experiment to determine if hemoglobin is degraded in the rumen to find possible false-positive tests due to blood from extra gastrointestinal sources and subsequently an observational study to estimate the diagnostic properties of the test with the observed lower detection limit. The observational study included primarily dairy cattle at slaughter, where we could observe the actual lesions postmortem. The detection limits of the tests marketed as Hemo-Fec^®^ and Hemoccult II^®^ SENSA^®^ were 1–2 mL blood/kg feces and 2–4.5 mL blood/kg feces, respectively. Hemoglobin was not degraded in ruminal fluid and could possibly bypass the rumen and be detected in feces. In the observational study, the Hemo-Fec^®^ test had no diagnostic value in dairy cattle with superficial erosions, with scarring, and with <4 acute or chronic lesions. The test had diagnostic potential in cattle with ≥4 acute or chronic lesions, where the proportion of true positives exceeded the proportion of false-positive results. However, many false-positive reactions make the use of the test a challenge.

**Abstract:**

Non-perforating abomasal lesions occur with a high prevalence in slaughtered dairy cattle. Ante mortem diagnosis is a challenge, but the presence of occult blood in feces is suggested as a diagnostic criterion. The lower detection limit of Hemo-Fec^®^ (Med-Kjemi, Asker, Norway) and Hemoccult II^®^ SENSA^®^ (Beckman Coulter, Brea, California, USA) for fecal occult blood were estimated. The Hemo-Fec^®^ and Hemoccult II^®^ SENSA^®^ could detect 1–2 mL and 2–4.5 mL of blood in 1000 g of feces, respectively. Therefore, the Hemo-Fec^®^ test was selected to access hemoglobin degradation in the rumen to establish if blood from outside the gastrointestinal tract could result in false-positive tests and an observational study to estimate the diagnostic sensitivity and specificity. Rumen microbiota did not degrade hemoglobin in a 1% blood concentration in vitro during 48 h of fermentation. The Hemo-Fec^®^ test was only able to detect cattle with ≥4 acute lesions (diagnostic sensitivity: 0.40 [95% confidence interval (95% CI): 0.32–0.48] and ≥4 chronic lesions (sensitivity: 0.44 [95% CI: 0.35–0.52]). The Hemo-Fec^®^ test had no diagnostic potential to detect superficial erosions or scar tissue in abomasa. Furthermore, the specificity was 0.71 [95% CI: 0.68–0.75%], and a positive test is thus not equivalent with abomasal lesions in cattle.

## 1. Introduction

Abomasal lesions at the time of slaughter have been reported with a prevalence of up to 84% in dairy cattle [1], 66% in fattening bulls [2], and up to 77% in veal calves [3]. All but a few are different types of non-perforating lesions, e.g., mucosal erosions, acute or chronic deeper lesions or scars [1,4,5,6]. Of the abomasal lesions, acute lesions have been reported with a prevalence between 62–66% and chronic lesions have a reported prevalence between 41–44% [1,6]. The impact on production and reproduction appears to be limited, but the lesions may still be painful to the affected animal [5,7]. However, ante mortem diagnosis of abomasal lesions is challenging. Although bleeding lesions should be detectable by testing for occult blood in feces, abomasal lesions are not the only sources of blood found in feces; other sources could be i.e., recent rectal examinations, epistaxis or traumatic lesions in the gastrointestinal tract. Besides, it is unknown whether abomasal lesions bleed continuously or periodically.

Few veterinary tests are available for detection of occult blood in feces within minutes. The only veterinary test for fecal occult blood we could acquire was the SUCCEED^®^ Equine Fecal Blood Test (Freedom Health LLC, Aurora, OH, USA). This is an immunochemical test for detection of albumin from undigested blood in the hindgut or hemoglobin in the foregut of equines. The cost of this test is approximately € 40 per test, which is too expensive for routine use in cattle.

Traditional fecal occult blood tests in humans have included the use of products with one of the following active compounds to detect hemoglobin: orthotolidine, benzidine or guaiac [8,9]. The common biochemical mechanism for all the substances is an oxidation of hemoglobin [10]. The highly sensitive orthotolidine- and benzidine-based tests were abandoned in the 1980s due to their carcinogenic effects [11,12]. The less carcinogenic benzidine derivate, tetramethylbezidine (TMB), has been studied for its ability to detect fecal occult blood [12,13]. TMB has been reported as having almost the same sensitivity for detection of blood as the benzidine tests [12]. A TMB test has been reported to have a detection limit at 3–5 mL blood per 100 g human feces with a sensitivity of 69% in 13 samples with a measured blood volume at 3–4.9 mL (mean 3.8 mL) per 100 g human feces. However, samples with lower amounts of blood were also found positive using the TMB test. The TMB test was positive in 5/30 of samples containing 1.0–1.9 mL (mean 1.5 mL) blood per 100 g human feces [14]. The Hemo-Fec^®^ (HF) (Med-Kjemi, Asker, Norway) test for fecal occult blood is based on TMB and is commercially available.

A recent study on cattle at slaughter used a guaiac test developed for human fecal samples [2]. Unfortunately, the production of this particular guaiac test has been discontinued. An alternative guaiac test is the Hemoccult II^®^ SENSA^®^ (HS) (Beckman Coulter, Brea, CA, USA). This test is reported with a detection limit as low as 1.6 mL of blood per 100 g human feces [15].

The HF test is currently produced with a listing price of € 0.11 per test, while the fecal occult blood guaiac test HS is produced with a listing price of € 1.30.

Blood or hemoglobin found in feces can have several origins i.e., bleeding throughout the entire gastrointestinal canal (e.g., from mouth wounds, lesions in the stomach or tears in the rectum), from a cow licking a bleeding wound or from epistaxis. The rumen microbiota is diverse and consists of many different microorganisms, and it could be speculated that the ruminal microbiota would degrade hemoglobin. However, we have not been able to find any scientific investigations of the possible degradation of hemoglobin.

The current study was conducted in series, where the results of two experiments were used in an observational study. First, the objective was to assess the detection limit of two tests for occult blood in feces (HF and HS). Then, we assessed if the test with the numerically lower detection limit was able to detect hemoglobin after ruminal fermentation, and finally an observational study to estimate the diagnostic sensitivity and specificity of the test for detection of different types of non-perforating abomasal lesions in dairy cattle was performed.

## 2. Materials and Methods

To address the objectives, two experiments were carried out and one observational study was conducted.

### 2.1. Experiment A

The detection limits of the two tests (HF and HS) for the detection of blood in bovine feces were determined based on titration. Feces from three clinically healthy cows (used for teaching at the Large Animal Hospital at University of Copenhagen) was collected before the morning sweeping. The cows were fed restricted concentrate and grass silage ad libitum. None of the cows had any rectal examinations performed in the weeks prior to the experimental study. Only manure with no visible contamination (e.g., bedding, straw, hoof prints, etc.) was collected. All fecal samples were tested for the presence of occult blood with the SUCCEED^®^ Test and the HF test. This was done to verify that the negative control samples were without hemoglobin. Only fecal samples that had tested negative with the two fecal occult blood tests were used for the experimental setup: The determination of the diagnostic blood detection limits of the HF and the HS tests.

Four batches consisting of 4000 g of feces were collected; Batches 1 and 2 were made with feces from two cows, and Batches 3 and 4 were made with feces from one cow. Each batch was subdivided into four plastic bags (sub-batches). A volume of fresh non-stabilized bovine whole blood was added to three of the four sub-batches as shown in Table 1. Feces and blood were mixed for 10 min to distribute the blood evenly in the feces. After the mixing, blood and feces appeared visually evenly distributed.

Three samples were collected from each sub-batch and tested with the HF and HS tests. The first test was done immediately following mixing. The second test was done eight hours (h) after the mixing, with the following tests done every 12 h until the last test 56 h after mixing. All sub-batches were stored at 5 °C between tests.

The HF test was used according to the instructions of the manufacturer: A pea-sized amount of feces was smeared on a piece of filter paper with a cotton swap. This was done separately for each sample and test. Then one drop of Reagent A immediately followed by one drop of Reagent B were added on top of each sample applied to the filter paper. The results were interpreted within 10 s after the drop of Reagent B. A bluish-greenish-yellowish color of the HF test appearing on the filter paper was considered a positive result. If none of the colors mentioned above were observed within 10 s after the drop of Reagent B had been added, the test was considered negative.

All the samples were also tested using the HS test. The HS test cards were prepared the day after the batches were mixed. The manufacturer’s instructions were followed, and a test card was made for each of the sub-batches. The test cards contain two test windows at the front and a shared test field at the back. Feces corresponding to approximately the size of a pea were smeared at each test window. The test card dried for at least 48 h at room temperature. To assess the tests, two drops of the included reagent were applied on each sample from the back and one drop was applied to the control patch for comparison of a positive or negative test. A blue color would appear if a test was positive. The control patch had a control for both a positive and a negative test. The results were evaluated and registered within 60 s after application of the reagent.

### 2.2. Experiment B

This experiment was carried out to determine if rumen microbes degrade hemoglobin. Rumen fluid was harvested before the morning feeding from two clinically healthy rumen cannulated non-pregnant and non-lactating Danish Jersey heifers. The heifers were fed a maintenance level of hay and served as teaching animals at the University of Copenhagen. The rumen fluid was filtered through two layers of household cheesecloth to remove the large feed particles. The pH value of the filtered rumen fluid was measured, and the rumen fluid was then mixed with a standard buffer solution consisting of 35 g/L NaHCO_3_ and 4 g/L NH_4_HCO_3_ in the ratio 1:2. From this mixture, 90 mL of sample was transferred into 100 mL glass containers, which served as fermentation chambers. Non-stabilized bovine whole blood in the volumes of 1, 5 and 10 mL was added to three fermentation chambers, respectively, and fermented for 48 h at 39.5 °C in a thermo shaker incubator (C. Gerhardt GmbH & Co, Königswinter, Germany). The fermentation was conducted anaerobically using a standardized protocol [16]. One fermentation chamber with no added blood, only containing rumen fluid and standard buffered solution, served as negative control.

After fermentation, the liquid of the four glass containers were poured into 100 mL plastic containers and tested for the presence of hemoglobin with the HF test. The filtrates were transferred to filter paper using a cotton swap. Both the filter paper and the cotton swap were tested with HF according to the manufacturer’s guidelines as described above. After 10 s, the tests were evaluated, and the results were registered.

### 2.3. Observational Study

Feces from 1553 cattle was collected during an abattoir survey studying abomasal lesions described in a previous publication [1]. After evisceration of the cattle, the intestines were further divided. The rectal ampulla was opened in the longitudinal direction. An incision of 10–30 cm was made with a scalpel between the sigmoid colon and the rectum. When the intestine curled back from the incision, a fecal sample was collected from an area away from the incision to avoid blood contamination from the environment and cut mucosa.

The fecal samples were analyzed using the HF test as described above. The analysis of each sample was performed on the day of feces collection (Day 0) and 2 days after collection (Day 2). The fecal samples were stored at 5 °C between test days.

Abomasal lesions were also recorded as previously described, and approximately the same sample of cattle was used [1]. Briefly, Type I lesions were scored as one of four subtypes: Ia: superficial erosions of the mucosa, sometimes only identifiable due to lack of rugae. Ib: lesions with local hemorrhage and a punched-out appearance, and where there were no obvious alterations of the mucosa near the lesions. Ic: lesions with a crater-like appearance, where the edges of the lesions were bulging and often detritus such as fibrin or other inflammatory products were found at the surface of the lesion. Id: lesions where the mucosa makes retention towards the lesion in a star-shape, or holes in the spiral mucosal folds [1,6].

### 2.4. Ethical Statement

Experimental procedures involving experimental animals were all approved by the National Committee on Animal Experimentation, Denmark and the local ethical committee on University of Copenhagen, Denmark.

### 2.5. Statistical Assessment

No analytical statistics were carried out for Experiments A and B, because the results were grouped specifically with no need for statistical tests. The detection limit was determined based on the lower concentration of blood detected for each test.

In the observational study, “cow” was used as the study unit, and the diagnostic sensitivity was estimated for each combination of lesion types as the proportion of cows being HF positive at test-day 0 or test-day 2, respectively, among those with a given lesion subtype combination. The diagnostic specificity was estimated as the proportion of HF negative cows among those without any lesions. Many cows had multiple lesions and multiple lesion subtypes, and to assess if this had an impact on the diagnostic sensitivity, a univariable logistic regression for each of the lesion subtypes Ia, Ib, Ic and Id were carried out initially, and then a multivariable logistic regression. These were done separately for each test day. The outcome was the result from the HF test (positive or negative) and the predictors were lesion subtypes, including two-way interaction terms in the multivariable regression models. The number of lesion subtypes Ia, Ib and Ic were categorized in 0, 1, 2, 3, and ≥4 lesions, while lesion subtype Id was only grouped in 0, 1 and ≥2 lesions based on the distribution of observations. The multivariable logistic model was reduced using the likelihood ratio test, and *p* < 0.05 was considered statistically significant. The analyses were carried out in R v. 3.5.1 [17] with the logistic regression done using the lm()-function. The R package called “tidyverse” was used to visualize the data and perform the descriptive statistics [18].

## 3. Results

Table 2 shows the HF results for determination of the detection limit in Experiment A. All samples containing blood in concentrations below 1 mL per 1000 g feces were negative and all samples with concentrations above 1 mL per 1000 g feces were positive in the HF test at different time points the samples were tested. At the concentration 1 mL blood per 1000 g feces, some results were positive, and some were negative. Hence, the diagnostic detection limit was between 1 and 2 mL per 1000 g feces for the HF test.

Table 3 shows the results from the HS titration. All samples with concentrations higher than 2 mL per 1000 g feces were positive and the rest were negative. Thus, the detection limit was between 2 and 4.5 mL per 1000 g feces for the HS tests.

**Figure 1:**
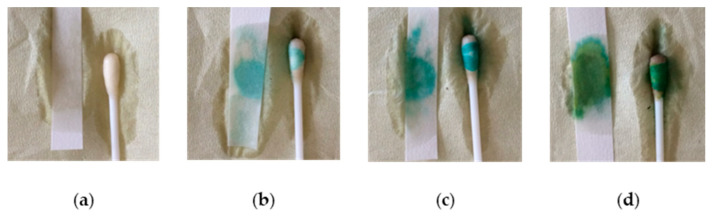
The four panels show the results of the Hemo-Fec^®^ test of fermented bovine whole blood in rumen fluid for a period of 48 h: (**a**) the negative control (0% blood/rumen fluid), (**b**) 1% blood/rumen fluid mix. (**c**) 5% blood/rumen fluid mix and (**d**) 10% blood/rumen fluid mix.

The filtrated rumen fluid had characteristic color changes correlating with the amount of added blood. The negative control was light brownish of color, whereas the three samples with 1, 5 and 10 mL of added blood had an increasing dark reddish-brownish-blackish discoloration when compared with the negative control. The pH of the rumen fluid ranged between pH 6.83 and 6.99 after the fermentation. The filtrate of the control with no added blood was negative for hemoglobin when tested using the HF test. The filtrates with added blood were all positive when tested with the HF reagents. Figure 1 illustrates the four test results.

Samples from 1553 cattle from 311 herds were included in the study. These included 1298 Holsteins, 147 crossbreeds, 84 Red Danish, 8 Hereford, 6 Danish Red Holstein, 5 Jersey, 2 Limousine, 2 Highland cattle and one cow of unknown breed. Of these, 298 were first parity cows, 410 were second parity cows, and 745 cows were of higher parities; 2 were bulls and the remaining 98 animals were heifers. Ten fecal samples were excluded due to not being tested at Day 2. Overall, 19% tested positive with the HF test at Day 0 and 34% tested positive at Day 2.

Table 4 shows the distribution of abomasal alterations and HF results. The proportion of positive HF samples was higher on Day 2, also for cattle with no lesions. The apparent diagnostic sensitivity was generally within the range of 16.6–21.3% on Day 0 and 27.1–43.5% at Day 2, except for cows with edema, of which there was only 15.

Table 5 shows the results of the univariable logistic regression. Only lesion subtypes Ib and Ic were associated with the HF result on Day 0 (*p* = 0.01 and *p* = 0.001, respectively), and on Day 2 (*p* = 0.0006 and *p* = 0.0001, respectively). Consequently, the HF had no diagnostic value for lesion subtypes Ia and Id, and only cattle with ≥4 lesions of subtypes Ib and Ic were significantly different from cattle with no lesions of the specific subtype. The diagnostic sensitivity (Se) and specificities (Sp) resulting from the estimates and standard errors (Table 4) were: Se = 0.25 (95% confidence interval (95% CI): 0.19–0.32) for detection of ≥4 subtype Ib lesions with Sp = 0.82 (95% CI: 0.80–0.85), and Se = 0.28 (95% CI: 0.21–0.36) for detection of ≥4 subtype Ic lesions with associated Sp = 0.83 (95% CI: 0.81–0.86). No multivariable associations existed for Day 0, as the model was reduced to results identical to the univariable model for lesion subtype Ic.

Table 6 shows the multivariable results for Day 2. Overall, only cattle with ≥4 lesions were identified with results deviating from the baseline for both lesion subtypes Ib and Ic. Cattle with ≥4 subtype Ib lesions had 1.7 times higher odds of testing positive in the HF test on Day 2 (95% CI: 1.4–1.9) and cattle with ≥4 subtype Ic lesions had 1.9 times higher odds of testing positive (95% CI: 1.6–2.3). The corresponding Se and Sp based on the multivariable regression estimates (Table 6) were: Se for detection of ≥4 subtype Ib lesions: 0.40 (95% CI: 0.32–0.48), Se for detection ≥4 subtype 1c lesions: Se = 0.44 (95% CI: 0.35–0.52), and Sp = 0.71 (95% CI: 0.68–0.75).

## 4. Discussion

The HF test had a detection limit of 1–2 mL blood per 1000 g of feces, which was lower than for the HS (2–4.5 mL blood per 1000 g of feces). Consequently, the HF test was selected for further work in the observational study. Rumen microbes did not appear to break down substantial amounts of hemoglobin when incubated for 48 h. Lesion subtypes Ia and Id, and <4 lesions of subtypes Ib and Ic were not associated with a positive HF-result. Only detection of ≥4 lesions of subtypes Ib and Ic were associated with positive test-results. The results thus showed that the high proportions of false-positive reactions (17–18% on Day 0 and 29% on Day 2) exceeded the true positive reactions for all but the cows with many subtype Ib or Ic lesions.

Several veterinary sources describe the use of a diagnostic fecal occult blood test in the pursuit of a diagnostic test for bleeding in the intestinal tract [2,19,20,21]. Three papers out of the four used or mentioned the use of the commercial orthotolidine tablet called Hematest (Ames Co, Elkhart, IN, USA). An example of this could be the evaluation of two commercial tests for fecal occult blood (the Hematest and the commercial guaiac test Hemoccult) and a non-commercial guaiac tincture by Payton and Glickman [21]. They concluded that the non-commercial guaiac tincture was the most sensitive test for fecal occult blood in vitro with a detection limit of 3.3 mL per 1000 g bovine feces. The Hematest was reported with a detection limit of 5.9 mL blood per 1000 g bovine feces, and the Hemoccult test was reported with a detection limit of 9.1 mL per 1000 g bovine feces, both in vitro. Their study also showed that most fecal samples were positive 7–9 h past infusion with the blood directly into the abomasum with both the Hematest and the Hemoccult tests.

The detectable concentrations of 0.2% and 0.45% for the HF and HS tests, respectively, correspond to a blood loss of 60 mL or 135 mL of blood if one presumes a cow excrete 30 kg of feces each day and the blood is evenly distributed in the daily fecal bolus. With this premise, we could assume that a cow weighs 800 kg of which 7% is blood. This corresponds to a blood volume of 56 L. Then, 60 mL of 56 L are approximately 0.1% of the total blood volume and 135 mL corresponds approximately to 0.2% of the total blood volume would be lost during 24 h. We assume abomasal lesions bleed intermittently, and not necessarily constantly. Then the blood will not be distributed evenly in the feces excreted during 24 h. To diagnose this intermittent bleeding with the fecal tests, testing must be performed several times during the day. A study performed by Rosenfield and colleagues [22] supports this assumption. The amount of blood being lost from non-penetrating abomasal ulcers are unknown. The results of Experiment A showed that the HF test had a lower detection limit than the HS test for detecting blood in feces in an experimental setting. We could have made further explorations of the exact volume to which the HF and HS tests could no longer detect blood in the 1000 g fecal samples. However, we did not pursue the minute amount of detectable fecal occult blood due to practical reasons. We were satisfied with the detection limit of 1–2 mL blood per 1000 g feces for the HF test and with the lower cost per test than the HS test, the HF test was used for the observational study. However, we also observed a relatively high proportion of false-positives (20.8% and 27.1% at the same day of collected feces and two days post collection, respectively) in the animals with no observed abomasal lesions. It is unknown whether these animals had been subject to a rectal examination before they were sent for slaughter. Many Danish dairy cows that are sent for slaughter have a rectal examination done to ensure they are not within the last month of gestation. Generally, the proportion of positive HF samples compared to the lesion types found in the abomasa of the animals were at 16.6–21.3% at Day 0 and noticeably higher at Day 2 with 32.5–43.5% when the few animals (n = 15) with edema were excluded.

Cows with ≥4 lesions of subtype Ib or Ic were more likely to test positive (*p* < 0.01 and *p* < 0.001, respectively). Since both lesion subtypes are expected to be bleeding, these results support our expectations. Neither the size nor the site of the lesions was taken into consideration. One could speculate that a large lesion would bleed more than several smaller ones, and with a bleeding lesion located closer to the aboral part of the abomasum, the blood would be less affected by digestive juices than a lesion located in the most oral part of the abomasum. If the hemoglobin is not broken down by the digestive juices of the abomasum, the location of a lesion would not have an impact on the HF test result of the feces. Nonetheless, our results showed a clear association between having many potential bleeding lesions (subtypes Ib and Ic) and the detection of blood in the feces. Furthermore, our results showed a higher sensitivity when samples are stored for two days. This could be an indication that blood degrades in the fecal matter when stored cold for two days, releasing the hemoglobin from the erythrocytes and thereby giving a quicker positive reaction with smaller quantities of blood in the sample. This is supported by the findings of Payton and colleagues, who found that the probability of a positive test increased when their samples were stored at 5 °C for two days [21]. Alternatively, it could be an indication of the effect of plant oxidases that yields a false-positive reaction. TMB is known to react with several different oxidases, e.g., broccoli, yellow corn, cucumber, mushroom, onion, etc. [23]. However, false-positive tests were not experienced in Experiment A. All samples except one were either positive or negative at all six tests within a test period of up to 56 h. A likely explanation is that 1 mL/1000 g is just around the detection limit, where minute differences in concentrations can yield different results.

Smith and colleagues [20] also used the Hematest as a diagnostic test for abomasal lesions. They found the diagnostic sensitivity to be 0.77 (20 test-positive among 26 cattle with abomasal lesions including 15 cattle with penetrating lesions and a total of 296 head of cattle) and a diagnostic specificity of 0.97 (with 8 false-positive Hematests of 270 ulcer-negative cattle in a total population of 296 head of cattle). However, the study by Smith and colleagues was based on hospitalized cows and was not a slaughter population like our study, and spectrum bias occurred due to the distribution of lesions, which included not only non-penetrating lesions, but also penetrating lesions.

Hund and colleagues [2] used the TMB test Combur Test (Roche Diagnostics GmbH, Vienna, Austria) as a reference for blood and the guaiac test Hemo FEC (Roche Diagnostics Gmbh, Vienna, Austria) as their diagnostic test. They reported a Se of Hemo FEC of 13% (95 CI: 0.035–0.36) and a Se of Combur Test of 63% (95% CI: 0.39–0.82), with corresponding specificities of 0.94 (95% CI: 0.73–0.99) and 0.50 (0.28–0.72) in relation to abomasal lesions. Their findings showed the TMB test to be more sensitive than the guaiac test, which supports our findings in Experiment A and B, but also that the test was more unspecific. However, their findings were based on a limited population of 33 cows, 17 without lesions and 16 with Type I lesion subtypes Ia, Ib and Ic.

An overall difference between Experiment A and the observational study was observed. In Experiment A we mixed feces with different quantities of fresh whole blood, whereas the blood in the feces in the observational study must have went through some digestive processes, if the blood originated from the abomasum or from the upper gastrointestinal tract. Even though we showed that fermentation in rumen fluid did not break down larger quantities of hemoglobin, it is not unlikely that the digestive processes of the gastrointestinal tract makes the hemoglobin more accessible for the HF test. Another explanation could be that the HF test gave a false-positive reaction due to other substances in the bovine fecal matter. The cows donating feces in Experiment A were situated at the Large Animal Hospital at University of Copenhagen, which provides different types of feed than what are typically provided for the dairy breeds in the stables. The hospital fed the cows grass silage ad libitum and restricted concentrate, whereas most Danish dairy cows are fed a total mixed ration with large quantities of corn silage. Cox [23] showed that TMB has a positive reaction with specific plant oxidases in for example yellow corn. However, if the HF indeed reacted with the corn residues in the bovine feces, the proportion of positive results would be expected to be a lot higher since most included cows from the slaughterhouse were of dairy cattle, which are most often fed corn silage. However, the majority of animals did not provide positive results as shown in Table 5. It would not have been possible to determine the specific ration given to the individual cows in the observational study.

Hemoglobin has been demonstrated to have a relatively low absorption through the intestinal mucosa in rats and dogs (2.5% and 30%, respectively) [24,25] and have a low degree of both absorption and degrading through the gastrointestinal tract in humans [8,26]. These three species are all omnivorous and it must be expected that some of their iron uptake originate from ingested hemoglobin. The authors were not able to identify any literature supporting this in ruminants. However, it was demonstrated that the hemoglobin did not break down considerably when incubated for 48 h in rumen fluid. Smaller amounts of blood than tested in experimental study B might be relevant, when the primary interest was to determine whether blood would be degraded in rumen fluid or not. If lower concentrations should be detected in vitro, a titration test setup for blood in rumen fluid should be set up to determine the lowest possible detection range of blood in rumen fluid before incubation, but this was not pivotal to our study. However, if quantities of blood were able to pass the ruminal fluid without being degraded and cause false-positive results when testing for fecal occult blood it would have to be in quite large quantities. The most sensitive test (HF) could not detect volumes lower than 2 mL per 1000 g feces and we speculate that the quantity of ingested blood should be rather large to avoid a dilution in the rumen fluid within the detection limit of 2 mL blood per 1000 g feces. Various quantities of blood could be presented to the gastrointestinal system via, e.g., epistaxis, bleedings from the lungs, teething, licking a bleeding wound or other smaller lesions in the mouth, esophagus and upper or lower airways.

Likewise, any rectal examinations without sufficient amounts of rectal gel or gentleness could result in minor lesions or tears in the rectal mucosa that might give a false-positive result in regard of the diagnostic sensitivity of the HF test of abomasal lesions. Additionally, the observational study was conducted in a slaughterhouse, where blood was present all over, and the risk of contaminating the fecal samples with blood were quite high.

Although a relatively high number of false-positive results were seen in the observational study, it must be assumed that some of the positive tests are positive because of hemoglobin originating from the gastrointestinal tract, whether due to ulcerations or rectal examinations. Some of the false-positives could also be caused by a reaction between the HF reagents and some plant oxidases in the feces. As mentioned, Danish dairy cows consume large quantities of corn silage, which potentially could explain some false-positive results. However, if degraded corn from the ration should be the main contributor, we would expect a higher overall risk of testing positive. The manufacturer’s guidelines warned that oxygen from the air act as an oxidizer for the TMB reagent and can give a positive result. However, this should be compensated for by using the short period of time (≤10 s), where the test is recorded as positive or negative, nonetheless, no false-positive reactions were recorded in Experiment A.

A positive sample collected in a farm situation would be expected to have a higher diagnostic reliability than the samples collected at the slaughterhouse, because blood would normally not contaminate the environment at the farms as it does a slaughterhouse. In addition, the veterinary practitioner and the farmer usually know if they have performed a rectal examination prior to testing for fecal occult blood in a cow.

## 5. Conclusions

In conclusion, rumen microbiota did not break down hemoglobin beyond recognition in a 1% concentration of blood in rumen fluid. The HF test had a high analytical sensitivity in vitro. In vivo HF can detect multiple lesions of the bleeding lesion subtypes Ib and Ic. However, there is a high risk of a false-positive results, and caution should be made when proceeding to decision-making following the diagnostic process.

## Figures and Tables

**Table 1:** An overview of the in vitro titration experiment for identification of the detection limit of two fecal occult blood tests. Batches 2 and 3 were similar in volumes of blood, and they were made to investigate if pooling the fecal matter from different cows had an impact on the results.

Batch Number	Amount of Blood (mL) Added to Each Sub-batch (1000 g of feces)			
1	5	25	50	0
2	0.5	2	4.5	0
3	0.5	2	4.5	0
4	0.25	0.75	1	0

**Table 2:** The experimental in vitro setup testing the analytical detection limit of bovine blood in bovine fecal matter in different titrations tested with Hemo-Fec^®^. The letters A, B and C are the repeated samples made from each mixture of feces and whole blood (sub-batches), and the letters “N” and “P” represents the negative and positive samples, respectively. Batches 1 and 2 were comprised of feces from two cows whereas batch 3 and 4 were made with feces form one cow. Batch 2 and 3 were equal in both blood volumes and results, hence they were shown as one result. Likewise, the four sub-batches with 0 mL of blood added are shown as one result.

mL Blood per 1000 g of Feces [Batch Number]	Sample	Time (hours) after Mixing Feces and Blood					
		0	8	20	32	44	56
0	A	N	N	N	N	N	N
	B	N	N	N	N	N	N
[1, 2, 3, 4]	C	N	N	N	N	N	N
0.25	A	N	N	N	N	N	N
	B	N	N	N	N	N	N
[4]	C	N	N	N	N	N	N
0.5	A	N	N	N	N	N	N
	B	N	N	N	N	N	N
[2, 3]	C	N	N	N	N	N	N
0.75	A	N	N	N	N	N	N
	B	N	N	N	N	N	N
[4]	C	N	N	N	N	N	N
1	A	P	N	P	N	N	N
	B	P	N	P	N	N	N
[4]	C	P	N	N	P	N	N
2	A	P	P	P	P	P	P
	B	P	P	P	P	P	P
[2, 3]	C	P	P	P	P	P	P
4.5	A	P	P	P	P	P	P
	B	P	P	P	P	P	P
[2, 3]	C	P	P	P	P	P	P
5	A	P	P	P	P	P	P
	B	P	P	P	P	P	P
[1]	C	P	P	P	P	P	P
25	A	P	P	P	P	P	P
	B	P	P	P	P	P	P
[1]	C	P	P	P	P	P	P
50	A	P	P	P	P	P	P
	B	P	P	P	P	P	P
[1]	C	P	P	P	P	P	P

**Table 3:** In vitro test of the analytical detection limit of the Hemoccult II^®^ SENSA^®^ test using bovine feces and blood. The letters A, B and C are the repeated samples made on each mix of feces and whole blood (sub-batches). The letters “N” and “P” represents the negative and positive test results, respectively. Batches 1 and 2 were comprised of feces from two cows whereas Batches 3 and 4 were made with feces from one cow. Batches 2 and 3 were equal in both blood volumes and results, hence they were shown as one result. Likewise, the four sub-batches with 0 mL of blood added to the feces are shown as one result.

mL Blood per 1000 g of Feces [Batch Number]	Sample	Test Card	
		Left well	Right well
0	A	N	N
	B	N	N
[1, 2, 3, 4]	C	N	N
0.25	A	N	N
	B	N	N
[4]	C	N	N
0.5	A	N	N
	B	N	N
[2, 3]	C	N	N
0.75	A	N	N
	B	N	N
[4]	C	N	N
1	A	N	N
	B	N	N
[4]	C	N	N
2	A	N	N
	B	N	N
[2, 3]	C	N	N
4.5	A	P	P
	B	P	P
[2, 3]	C	P	P
5	A	P	P
	B	P	P
[1]	C	P	P
25	A	P	P
	B	P	P
[1]	C	P	P
50	A	P	P
	B	P	P
[1]	C	P	P

**Table 4:** Descriptive results of Hemo-Fec^®^ (HF) on slaughtered cattle the same day the fecal samples were obtained (Day 0) and two days after fecal sampling (Day 2). Cow was the study unit, but the individual cow may occur in multiple groups, e.g., group “Ia” includes all cattle with subtype Ia lesions along with those with other subtypes, while group “Ia + Ib” includes all cattle with at least Ia and Ib lesions. The diagnostic sensitivity with the corresponding confidence interval (CI) for both days are shown.

Lesion Subtype (LS) or Alteration	N ^1^	HF Test Results Day 0				HF Test Results Day 2			
		HF+ ^2^	HF− ^3^	P(HF+|LS) ^4^	CI ^5^	HF+ ^2^	HF− ^3^	P(HF+|LS) ^4^	CI ^5^
No lesions	96	20	76	0.208	[0.127–0.290]	26	70	0.271	[0.182–0.360]
Ia	661	110	551	0.166	[0.138–0.195]	215	446	0.325	[0.290–0.361]
Ib	503	102	401	0.203	[0.172–0.233]	192	311	0.382	[0.339–0.424]
Ic	760	155	605	0.204	[0.173–0.234]	283	477	0.372	[0.338–0.407]
Id	108	23	85	0.213	[0.182–0.244]	47	61	0.435	[0.342–0.529]
Ia + Ib	955	178	777	0.186	[0.157–0.216]	335	620	0.351	[0.321–0.381]
Ia + Ic	1138	213	925	0.187	[0.157–0.217]	399	739	0.351	[0.323–0.378]
Ia + Id	721	124	597	0.172	[0.143–0.201]	243	478	0.337	[0.303–0.372]
Ib + Ic	997	197	800	0.198	[0.167–0.228]	365	632	0.366	[0.336–0.396]
Ib + Id	568	117	451	0.206	[0.175–0.237]	219	349	0.386	[0.346–0.426]
Ic + Id	797	160	637	0.206	[0.175–0.237]	297	500	0.373	[0.339–0.406]
Ia + Ib + Ic	1267	239	1028	0.189	[0.159–0.218]	447	820	0.353	[0.326–0.379]
Ia + Ib + Id	991	186	805	0.188	[0.158–0.217]	351	640	0.354	[0.324–0.384]
Ia + Ic + Id	1553	288	1265	0.187	[0.163–0.211]	523	1030	0.352	[0.323–0.381]
Ib + Ic + Id	1019	200	819	0.196	[0.166–0.227]	371	648	0.364	[0.335–0.394]
Ia + Ib + Ic + Id	1278	241	1037	0.189	[0.159–0.218]	451	827	0.353	[0.327–0.379]
Edema	15	8	7	0.533	[0.281–0.786]	9	6	0.600	[0.352–0.848]
Hyperplasia	855	152	703	0.178	[0.152–0.203]	282	573	0.330	[0.298–0.361]
Nodules	499	105	394	0.210	[0.175–0.246]	198	301	0.397	[0.354–0.440]
Scar	58	12	46	0.207	[0.103–0.311]	22	36	0.379	[0.254–0.504]
SP_HF0_^6^	0.792								
SP_HF2_^7^	0.729								

^1^ N: the number of animals in the category, please note that one cow can have several lesion subtypes present at the same time and therefore one cow can be registered at more than one level; ^2^ HF+: the number of positive HF samples; ^3^ HF−: the number of negative HF samples; ^4^ P(HF+|LS): Diagnostic sensitivity regarding the category, except for No lesions, where it corresponds to 1 − Sp; ^5^ CI: 95% confidence interval of the diagnostic sensitivity; ^6^ SP_HF0_: the specificity of the HF test in regard of abomasal lesions and alterations at day 0; ^7^ SP_HF2_: the specificity of the HF test in regard of abomasal lesions and alterations at Day 2.

**Table 5:** Univariable fixed effects of abomasal lesion subtype and number of abomasal lesion subtypes on the Hemo-fec^®^ (HF) test at Day 0 and Day 2.

Variable	Level	N ^1^	HF Test Results Day 0			HF Test Results Day 2		
			Estimate ^2^	SE ^3^	*p*-Value	Estimate ^2^	SE ^3^	*p*-Value
Lesion subtype Ia	0 lesions (intercept)		−1.389	0.084	<0.0001	−0.640	0.070	<0.0001
	1	322	−0.305	0.175	0.082	−0.172	0.140	0.22
	2	142	−0.060	0.230	0.79	0.240	0.185	0.19
	3	103	−0.460	0.299	0.12	−0.395	0.235	0.09
	≥4	94	0.016	0.270	0.95	−0.022	0.229	0.93
Lesion subtype Ib	0 lesions (intercept)		−1.536	0.081	<0.0001	−0.776	0.066	<0.0001
	1	168	−0.117	0.225	0.60	0.136	0.175	0.44
	2	101	0.074	0.267	0.78	0.185	0.218	0.40
	3	45	0.004	0.398	0.99	−0.019	0.329	0.95
	≥4	189	0.046	0.186	0.014	0.553	0.161	0.0006
Lesion subtype Ic	0 lesions (intercept)		−1.602	0.095	<0.0001	−0.835	0.077	0.0001
	1	315	−0.042	0.180	0.82	0.069	0.144	0.63
	2	182	0.236	0.207	0.26	0.318	0.172	0.06
	3	110	0.326	0.250	0.19	0.429	0.209	0.04
	≥4	153	0.663	0.203	0.001	0.691	0.180	0.0001
Lesion subtype Id	0 lesions (intercept)		−1.494	0.068	<0.0001	−0.711	0.060	0.0001
	1	66	0.270	0.301	0.37	0.405	0.255	0.11
	≥2	42	0.047	0.399	0.91	0.520	0.315	0.10

^1^ N: number of cows with the different number of lesion subtypes, also please note that one cow can have several lesion subtypes present at the same time and therefore one cow can be registered at more than one level; ^2^ Estimate of the probability of testing HF positive with the given amount of lesion subtypes; ^3^ SE: standard error of the estimate.

**Table 6:** Multivariable logistic regression of Hemo-Fec^®^ (HF) Day 2 test results in relation to abomasal lesion subtypes Ib and Ic.

HF Day 2	Level	Estimate ^1^	SE ^2^	*p*-Value
	0 lesions (intercept)	−0.911	0.086	<0.0001
Lesion subtype Ib	1	0.125	0.176	0.48
	2	0.191	0.220	0.39
	3	−0.071	0.332	0.83
	≥4	0.504	0.162	0.002
Lesion subtype Ic	1	0.043	0.145	0.77
	2	0.305	0.173	0.077
	3	0.395	0.211	0.061
	≥4	0.654	0.181	0.0003

^1^ Estimate of the probability of testing HF positive with the given number of lesion subtypes; ^2^ SE: standard error of the estimate.
